# A Truthful Incentive Mechanism for Online Recruitment in Mobile Crowd Sensing System

**DOI:** 10.3390/s17010079

**Published:** 2017-01-01

**Authors:** Xiao Chen, Min Liu, Yaqin Zhou, Zhongcheng Li, Shuang Chen, Xiangnan He

**Affiliations:** 1 Institute of Computing Technology, Chinese Academy of Sciences, No. 6 Kexueyuan South Road, Haidian District, Beijing 100190, China; chenxiao3310@ict.ac.cn (X.C.); zcli@ict.ac.cn (Z.L.); chenshuang@ict.ac.cn (S.C.); 2 School of Computer and Control Engineering, University of Chinese Academy of Sciences, No. 19 A Yuquan Road, Shijingshan District, Beijing 100049, China; 3 Source Clear, 20 Ayer Rajah Crescent, Singapore 139964, Singapore; yaqinchou@gmail.com; 4 School of Computing, National University of Singapore, Computing 1, Computing Drive, Singapore 117417, Singapore

**Keywords:** mobile crowd sensing system, online incentive, truthful mechanism, single-parameter mechanism

## Abstract

We investigate emerging mobile crowd sensing (MCS) systems, in which new cloud-based platforms sequentially allocate homogenous sensing jobs to dynamically-arriving users with uncertain service qualities. Given that human beings are selfish in nature, it is crucial yet challenging to design an efficient and truthful incentive mechanism to encourage users to participate. To address the challenge, we propose a novel truthful online auction mechanism that can efficiently learn to make irreversible online decisions on winner selections for new MCS systems without requiring previous knowledge of users. Moreover, we theoretically prove that our incentive possesses truthfulness, individual rationality and computational efficiency. Extensive simulation results under both real and synthetic traces demonstrate that our incentive mechanism can reduce the payment of the platform, increase the utility of the platform and social welfare.

## 1. Introduction

With abundant portable sensors (e.g., camera, compass, microphone, gyroscope, etc.) embedded in mobile devices (e.g., smart phones, wearable devices, tablets, etc.), people are available to collect sensing data when they roam in the city. Owing to the low deploying cost and high sensing coverage, numerous mobile crowd sensing (MCS) systems spring up to solve large-scale mobile sensing tasks, such as wireless signal strengths [1], traffic information mapping [2], air quality monitoring [3], parking [4], and so on.

In a typical MCS system, a cloud-based platform first divides sensing tasks into a set of unit sensing jobs. For example, collecting data for one location at one time slot can become a unit job for collecting sensing data in the city during a long time. Then, the platform publishes these jobs and takes rounds to make irreversible online decisions on user recruitment for every job before the deadline. Mobile users join and leave the system dynamically with pleasure. The selected users submit data to the platform after undertaking assigned sensing jobs.

Motivating users to participate is the key to the success of MCS systems. Since people are selfish in general, few mobile users voluntarily participate in sensing, especially considering the fees of uploading data through cellular networks and limited resources in smart phones, such as memory and energy. Consequently, MCS systems would fail without desirable sensing data from enough participants. To solve this problem, the design of incentive mechanisms is an effective approach via giving some rewards to users as compensations.

Besides the incentive mechanism, the quality of sensing service is crucial to the MCS system. As we know, inefficient sensing produces low quality sensing data, which harms the preciseness of MCS systems directly. To support the desired service, the platform shall select winners with high qualities of sensing service. Recently, some research works [5,6,7] have proposed auction-based incentive mechanisms with the consideration of the service quality. However, in these works, users’ sensing qualities are known to the system as prior information while determining winners.

However, the users’ service qualities are uncertain and unknown to the system during winner selections in practice. Since users move around and the wireless signals are not stable, users’ service qualities vary at times. Moreover, as users’ service qualities can be only calculated after submitting sensing data, the qualities are unknown to the platform while selecting winners. That is to say, service qualities are ex post information. Recently, some research works [8,9,10] have paid attention to the ex post service quality with the consideration of dynamic participation. However, their systems rely on users’ historical movement regulations to estimate users’ service qualities. Without this previous knowledge of users, these works cannot fit in a new MCS system. Besides, these works ignore users’ dishonest behaviors in overstating bidding prices, which results in more payment and less utility of the platform.

To solve the aforementioned problem, we propose a novel truthful incentive-based on online auction mechanism (TOAM) with the consideration of ex post service quality and dynamically-arriving users for a new MCS system without requiring previous knowledge of the users. In TOAM, the platform determines a winner according to the arriving users’ bidding prices and learned expected service qualities in every allocation round. As users are selfish, but rational in general, users may overstate their costs in their bids for higher payoff, which results in poor outcomes [11]. Hence, truthfulness and individual rationality are essential in auction-based incentives, where truthful costs are guaranteed in users’ bids and the payoff of winners is not negative. For the uncertain and unknown service quality, we believe users’ expected service qualities are fixed. Furthermore, one user’s service quality of one job is stochastically drawn from some unknown distributions. Therefore, the platform learns users’ expected service qualities and makes sequential decisions on winner selections with an exploration-exploitation trade-off. The exploration-exploitation is a balance between remaining with the best choice that can gain the highest profits once and exploring a new choice that might give higher profits in the future.

Calculating payment in truthful incentive mechanisms could be computationally impossible owing to the online restriction [12]. To achieve computational efficiency, we adopt a framework of designing a truthful-in-expectation mechanism with single-parameter users [12] in TOAM. Here, the user’s expected service quality is the single parameter. With random sampled bidding prices for winner selections and payment decisions, the key is to design a novel ex post monotonicity of the allocation rule of sensing jobs.

The major technical contributions in our paper are as follows.

- To the best of our knowledge, this is the first truthful incentive based on auction theory with consideration of ex post service qualities and dynamically-arriving users in a new MCS system without requiring previous knowledge of users. In TOAM, the platform learns users’ expected service qualities and makes sequential online decisions on winner selections with an exploration-exploitation trade-off.
- To achieve truthfulness with the consideration of computational efficiency in our situation, we adopt a framework proposed in [12] and design a novel ex post monotone allocation rule to select proper winners.
- We analyze if TOAM possesses truthfulness, individual rationality and computational efficiency theoretically. Besides, extensive simulation results on both real and synthetic traces verify the efficient of our incentive TOAM, which can decrease the payments, improve the utility of the platform and social welfare.

The rest of the paper is organized as follows. We introduce our system model, review some technical preliminaries and formulate our problem in Section 2. Our incentive TOAM is detailed in Section 3. Three attractive properties of TOAM are proven in Section 4. We evaluate TOAM and present the results in Section 5. Related work is reviewed in Section 6, and the conclusion is drawn in Section 7 finally.

## 2. System Model, Technical Preliminaries and Problem Formulation

### 2.1. System Model

**Figure 1:**
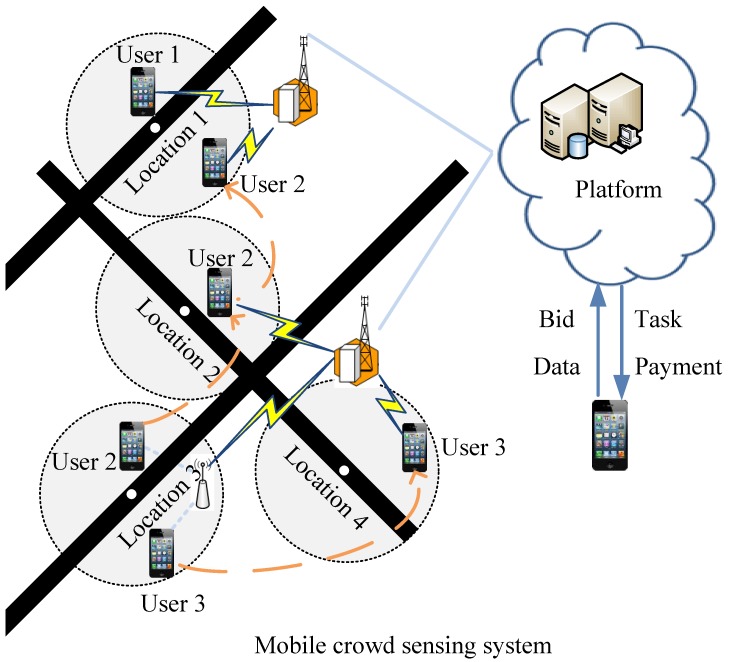
An example of the mobile crowd sensing system.

With an instance of MCS systems shown in Figure 1, we introduce our system model. The system consists of two types of entities, namely the platform residing in the cloud and a set of mobile phone users, denoted as N={1,2,...,n}. For the platform, the IaaSmodel of the cloud service presented in [13] can be used so as to run the platform in a trusted state. For the mobile phone users, they can download corresponding crowd sensing applications to participate in collecting sensory data.

To conduct some homogeneous sensing tasks, at first, the platform publishes a set of unit sensing jobs in order to collect sensory data from a set of locations $L=\{l_{1},l_{2},...\}$ before deadline *T*. Then, at any time slot t(t≤T), the platform selects a winner from a set of arriving users $N_{jt}\left(N_{jt}\subseteq N\right)$ at any location $l_{j}\left(l_{j}\in L\right)$ to execute a sensing job. To simplify, we assume users can complete one sensing job at their current locations within one slot. Since users roam in the city and participate in sensing activities intermittently, the available users for a job in location $l_{j}$ vary with time. Therefore, the platform should make sequential online decisions to allocate jobs. An the end of the time slot, the system pays the winner rational money after receiving sensing data.

To select appropriate users, both users’ costs and service qualities should be considered. The cost $c_{i}$ (i.e., $c_{\min}\leq c_{i}\leq c_{\max}$) of user *i* for one sensing job is private information, where $c_{\min}$ and $c_{\max}$ are known as the minimum and maximum cost. To calculate the cost, the application in a mobile phone can measure the cost of resources (e.g., computing resource and energy) for undertaking one unit sensing job. $c_{\max}$ and $c_{\min}$ are the maximum and minimum costs of resources in various mobile phones measured by the system before the mobile crowd sensing systems’ release. On account of different sensing devices equipped in mobile phones and users’ diverse behaviors, users have differential service qualities. We believe every user’s sensing ability is static, so we assume the expected service quality $q_{i}$ (i.e., $q_{\min}\leq q_{i}\leq q_{\max}$) of user *i* is fixed and also privately held by user *i*, where $q_{\min}$ and $q_{\max}$ are the minimum and maximum value, respectively. Users’ service qualities vary with time, so we assume that the service quality $q_{it}$ of user *i* at time slot *t* is a stochastic parameter following a fixed distribution on $\left[q_{\min},q_{\max}\right]$ with expectation $q_{i}$. There are many methods to reflect users’ service qualities, such as directly calculating the deviation of users’ sensory data from the ground truths [14], inferred from users’ data by utilizing algorithms proposed in [15,16,17] without ground truths, etc. However, as our auction-based incentive is not restricted to any particular methods of quality calculation, the details of these methods are out of the scope of this paper. Instead, we give some values to represent qualities directly, where a high quality value means good service quality.

A general assumption [18] is given in this paper about auction mechanisms, i.e., users are symmetric, independent and risk-neutral. That is to say, users have the same common knowledge, except their private information, and determine their bidding prices independently, so as to achieve their maximum utilities without worrying about risks.

### 2.2. Technical Preliminaries

We review some crucial solution concepts used in this paper.

**Definition** **1.** (A user’s utility at time slot t): At time slot t, the utility $u_{i}$ of user i∈N equals $u_{it}=p_{it}-c_{i}$ if user i is online and selected as a winner to execute one sensing job, where $p_{it}$ denotes the payment from the platform; otherwise, $u_{it}=0$.

**Definition** **2.** *(Platform’s utility): The utility of the platform is defined as Equation* (1)*, where α is a coefficient that transforms service quality to monetary reward, and $x_{it}=1$ (or 0) indicates that user i is chosen (or not) at time t.*

(1) $$u_{0}=\sum_{t\leq T}\sum_{l_{j}\in L}\sum_{i\in N_{jt}}\left(\alpha q_{i}-p_{it}\right)x_{it},\;where\;x_{it}=\left\{0,1\right\},\;\forall i\in N$$

**Definition** **3.** *(Social welfare): Social welfare of the mobile crowd sensing system is defined as Equation* (2)*:*

(2) $$\pi =u_{0}+\sum_{t\leq T}\sum_{l_{j}\in L}\sum_{i\in N_{jt}}u_{it}=\sum_{t\leq T}\sum_{l_{j}\in L}\sum_{i\in N_{jt}}\left(\alpha q_{i}-c_{i}\right)x_{it},\;where\;x_{it}=\left\{0,1\right\},\;\forall i\in N$$

**Definition** **4.** (Individual rationality): A mechanism satisfies the individual rationality constraints if the utility $u_{it}$ of user i is not negative at every time slot t before deadline T.

**Definition** **5.** (Ex-post monotonicity of the allocation rule [12]): An allocation rule is ex post monotone if increasing one user’s bidding price does not raise his or her probability of winning while keeping others’ bidding prices the same.

**Definition** **6.** (Truthful-in-expectation mechanism [12,19]): A mechanism is truthful-in-expectation if a risk-neutral user maximizes expected utility by adopting a truthful strategy in bidding, whatever the bids of others, where the expectation is taken with random coin flips of the mechanism.

**Definition** **7.** (Computational efficiency [20]): An incentive is computational efficiency if it takes polynomial time to produce the outcome.

### 2.3. Problem Formulation

As users’ service qualities are uncertain and unknown, the goal of the platform is to select winners with the largest expected service qualities and smallest costs to execute sensing jobs. Mathematically, this can be represented as follows:

(3) $$\begin{array}{c} \max\sum_{t\leq T}\sum_{l_{j}\in L}\sum_{i\in N_{jt}}q_{i}x_{it}\\ \min\sum_{t\leq T}\sum_{l_{j}\in L}\sum_{i\in N_{jt}}c_{i}x_{it}\\ s.t.\;x_{it}=\left\{0,1\right\},\;\forall N_{jt}\subseteq N \end{array}$$

There are two objectives in the above Equation (3): To maximize the sum of the expected service qualities of winners and to minimize the sum of the costs of winners. It is not easy to solve multi-objective programming; we take a common approach in optimization and convert this multi-objective programming to a single-objective programming. Then, the platform aims to select cost-effective workers with the highest expected price-quality ratios as follows.

(4) $$\begin{array}{c} \max\sum_{t\leq T}\sum_{l_{j}\in L}\sum_{i\in N_{jt}}(q_{i}/c_{i})x_{it}\\ s.t.\;x_{it}=\left\{0,1\right\},\;\forall N_{jt}\subseteq N \end{array}$$

We utilize the technique of designing a single-parameter mechanism to solve the above problem. Though two parameters—cost and expected service quality—should be considered in allocations, users’ costs can be revealed in their bidding prices if truthfulness is guaranteed. Thus, the expected service quality is the single-parameter in the above problem. However, in order to guarantee the deterministic truthfulness, it is costly or impossible to calculate winners’ payments by adopting the Vickrey–Clarke–Groves (VCG) auction due to online constraints in MCS systems. Therefore, to satisfy computational efficiency, our goal turns into the design of a truthful-in-expectation mechanism with the consideration of dynamically-arriving users and their unknown service qualities.

For the single-parameter mechanism, Babioff et al. [12] propose a generic framework in which an ex post monotone allocation rule can be transformed into a truthful-in-expectation mechanism via involving random perturbation to users’ bidding prices. To achieve our goal, we aim to design a novel ex post monotone allocation rule that can be transformed into a truthful-in-expectation mechanism by utilizing the transformation proposed in [12].

Given a monotone allocation rule A and a parameter μ∈(0,1), we describe the transformation procedure proposed in [12] with our situation so as to realize a truthful-in-expectation mechanism.

1. User *i* submits his or her bidding price $b_{i}$ (i.e., $b_{i}\leq c_{\max})$ when he or she participates in the system for the first time.
2. The platform computes user *i*’s new bidding price ${\tilde{b}}_{i}$ for allocation as follows. With probability 1−μ, ${\tilde{b}}_{i}=b_{i}$; else, ${\tilde{b}}_{i}=b_{i}\beta^{1/(1-\mu )}+c_{\max}\left(1-\beta^{1/(1-\mu )}\right)$,where β∈(0,1) is picked uniformly at random.
3. For every time slot *t*, the platform assigns sensing jobs to users for every sensing location $l_{j}$ according to the allocation rule $A({\tilde{B}}_{jt})$, where ${\tilde{B}}_{jt}$ consists of new bidding prices for all users in $N_{jt}$.
4. The platform calculates users’ payments as follows. For every selected user *i*, $p_{it}=b_{i}$ if ${\tilde{b}}_{i}=b_{i}$; otherwise $p_{it}=b_{i}+1/\mu \left(c_{\max}-b_{i}\right)$. For any other unselected user *k*, $p_{kt}=0$.

Table 1 lists frequently-used notations.

## 3. Incentive Mechanism Design

### 3.1. System Overview

**Figure 2:**
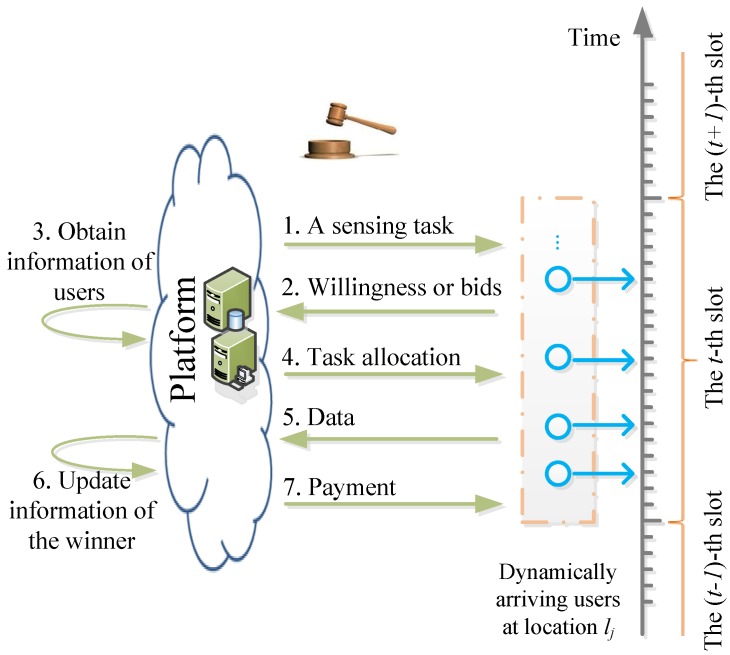
Our incentive framework for a mobile crowd sensing system with dynamic users and uncertain service quality.

For simplicity, we assume that there is one sensing task that needs to collect data from all sensing locations *L* during *T* time slots. The platform divides the tasks into a set of spatio-temporal sensing jobs. For example, *T* sequential sensing jobs are required to be allocated for each location $l_{j}\left(l_{j}\in L\right)$. Since users move around and participate in sensing intermittently, the platform requires selecting winners irrevocably in sequential auction rounds. Take one auction round for a job at sensing location $l_{j}$ during time *t* as an example: TOAM includes seven steps, as shown in Figure 2. We describe the procedure of our mechanism briefly as follows:

1. The platform announces the sensing job when users arrive at location $l_{j}$.
2. If users are new in the system, they create and submit their optimal bidding prices for this job to the platform independently. Otherwise, users are just required to show their willingness of participation.
3. The platform obtains users’ information for this allocation. If users are new, the platform gets and records their bidding prices from their bids. Otherwise, the system obtains users’ information from the storage, including users’ bidding prices, estimated expected service qualities, and so on.
4. Based on users’ new bidding prices perturbed by the platform randomly and users’ expected service qualities observed by the platform, the platform selects a winner to perform this job.
5. At the end of time *t*, the winner submits sensory data to the platform.
6. The platform updates the information of the winner if necessary according to the allocation rule.
7. The platform pays the winner with a rational price.

### 3.2. Allocation Algorithm

As we described before, the key of TOAM is to design an ex post monotone sensing task allocation algorithm. In reality, users who compete for one job vary over time. In order to realize the ex post monotone, the allocation algorithm should be designed carefully to control the influence of the liar who overstates his or her bidding price. The main idea of our algorithm can be reduced to four points principally.

1. For one job, construct an active set of users according to a bidding bound (reserve price) for a later random selection. In order to decrease the winning probability of the liar, we select a bidding bound from the arriving bidding prices with some probabilities. If users’ bidding prices are higher than the bidding bound, remove these users from the set of arriving users. The rest of arriving users construct the active set. To avoid removing too many users at the beginning of the system, the probability of selecting a low bidding bound increases with the passage of time.
2. With the increasing updated number, we estimate users’ expected service qualities in the non-decreasing trend. By doing so, it reduces the uncertain influence of being selected on the learned expected service qualities.
3. Update winners’ information (including the expected service qualities) if they are selected randomly. Due to the liar’s overstated bidding price, some others may win instead of the liar. We call these winners direct-winners. These direct-winners’ expected service qualities may be improved in this way. Thus, the liar’s winning probability can be reduced to a certain extent in the future.
4. Do not update winners’ information if they are selected according to the price-quality ratios. If winners are selected according to price-quality ratios, the updated information of direct-winners may affect the results. However, as winners’ information remains the same in this way, the direct-winners will not disturb others more. Therefore, the influence of the liar is controlled.

The pseudocode of the allocation algorithm in TOAM is shown in Algorithm 1. We take an allocation round at location $l_{j}$ during time slot *t* as an example.

If there is no user for this allocation round, the platform does nothing. Otherwise, in Algorithm 1, there are three cases of being selected from the set $N_{jt}$ of available users, which are random selection for initialization, random selection with the bidding bound limitation and greedy selection according to the price-quality ratios. By these random selections, users will not leave MCS systems since they have not won sensory jobs for a long time. We use C1, C2 and C3 to represent each case, respectively.

- C1: We randomly select a new user as the winner from $N_{jt}$. We call users who have joined the system, but have not undertaken sensory jobs new users. The platform updates the winner’s corresponding information with the observed winner’s actual service quality at the end of time slot *t* by calling updating Algorithm 2 (UpdateInformationofWinner).
- C2: The platform selects the winner randomly with the bidding bound limitation. There are three steps. Firstly, we randomly designate a user from $N_{jt}$. Secondly, construct an active set $S_{jt}$ of users with the bidding bound limitation. Thirdly, if the designated user is in the active set $S_{jt}$, he or she is the winner. Then, update the winner’s corresponding information at the end of time slot *t* by calling updating Algorithm 2 (UpdateInformationofWinner).
- C3: If the randomly-designated user is not in $S_{jt}$, the platform optimistically picks the user with the highest estimated price-quality ratio among the users in $S_{jt}$.

**Algorithm 1:** Allocation algorithm.
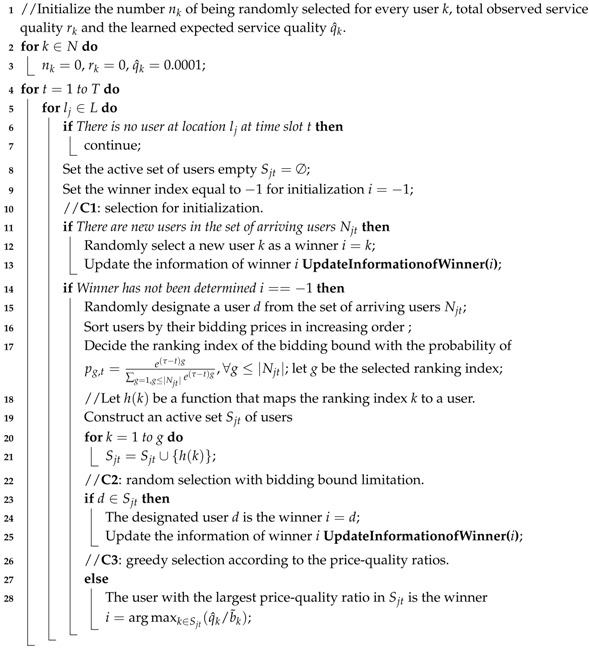


**Algorithm 2:** UpdateInformationofWinner.**1** //**Input:** user *i*; **2** Update the number of being randomly selected $n_{i}=n_{i}+1$; **3** Add the observed service quality $r_{it}$ to the total service quality $r_{i}=r_{i}+r_{it}$; **4** Update the learned expected service quality ${\hat{q}}_{i}=\max(\frac{r_{i}}{n_{i}}+\sqrt{\frac{8\ln t}{n_{i}}},{\hat{q}}_{i}$);

## 4. Theoretic Analysis

To prove the ex post monotonicity of our allocation algorithm, we introduce some definitions at first. Assume user *i* overstates the bidding price. Let $B=(b_{-i},b_{i})$ denote the bid vector of all users, where $b_{i}$ is user *i*’s bid and $b_{-i}$ denotes others’ bids. Let $B^{+}=\left(b_{-i},b_{i}^{+}\right)$ be the alternative bid vector, where $b_{i}^{+}>b_{i}$, but the others’ bidding prices remain the same as in *B*. ${\hat{q}}_{it}\left(B\right)$ and ${\hat{q}}_{it}\left(B^{+}\right)$ are defined as the learned expected service quality of user *i* at time slot *t* with the bid vector *B* and the bid vector $B^{+}$, respectively. Let $P_{it}\left(B\right)$ and $P_{it}\left(B^{+}\right)$ represent the probability that user *i* is chosen at time *t* with the bid vector *B* and the bid vector $B^{+}$, respectively.

**Lemma** **1.** At any end of time slot t, the learned expected service quality ${\hat{q}}_{it}\left(B\right)$ of user i with bid vector B is not lower than ${\hat{q}}_{it}\left(B^{+}\right)$ with bid vector $B^{+}$, that is ${\hat{q}}_{it}\left(B\right)\geq {\hat{q}}_{it}\left(B^{+}\right)$.

**Proof.**  From the allocation algorithm, we can see, at any time *t*, users’ expected service qualities may be updated when they are selected only in case C1 and C2. Now, we use mathematical induction to prove this lemma.

At time t=0, the case C1 is the only possible case of being chosen since all users are new in the system. Therefore, the probability of being chosen is the same with different bid vectors, that is $P_{i0}\left(B\right)=P_{i0}\left(B^{+}\right)$. Therefore, ${\hat{q}}_{i0}\left(B\right)={\hat{q}}_{i0}\left(B^{+}\right)$, and the lemma is true at the end of time slot t=0.

As shown in the algorithm, users’ service qualities remain the same with their last appearances in the system if they quit the system temporarily. Furthermore, users’ service qualities can be updated only when they are in the system. Therefore, we mainly describe the time slots when user *i* joins the system. Assume the lemma is true at end of time $t_{k}-1\left(t_{k}-1\geq 0\right)$. Now, we need to show that the lemma is also true at the end of time $t_{k}$, where user *i* is in the system at time $t_{k}$.

- C1: It is easy to see $P_{it_{k}}\left(B\right)=P_{it_{k}}\left(B^{+}\right)$ with different bid vectors if he or she is new in the system at $t=t_{k}$. Therefore, the lemma is true.
- C2: User *i* can be selected if user *i* is designated and stays in the active set. Now, we analyze user *i*’s probability of satisfying these two conditions. (1) As the random designated user is irrelevant with respect to his or her bidding price, user *i*’s probability of being designated is not related to the bid vector. (2) The selection of the ranking index bound is independent of the users’ bidding prices. Therefore, the ranking index bound is the same with different bid vectors. Since $b_{i}<b_{i}^{+}$, the ranking index of user *i* with bid vector *B* is not larger than that with bid vector $B^{+}$. Therefore, user *i*’s probability of staying in the active set with bid vector *B* is not lower than that with bid vector $B^{+}$. To sum up, $P_{it_{k}}\left(B\right)\geq P_{it_{k}}\left(B^{+}\right)$. Since the service quality increases with the updated number and ${\hat{q}}_{i(t_{k}-1)}\left(B\right)\geq {\hat{q}}_{i(t_{k}-1)}\left(B^{+}\right)$, we can get ${\hat{q}}_{it_{k}}\left(B\right)\geq {\hat{q}}_{it_{k}}\left(B^{+}\right)$.

Therefore, ${\hat{q}}_{it_{k}}\left(B\right)\geq {\hat{q}}_{it_{k}}\left(B^{+}\right)$. To sum up, ${\hat{q}}_{it}\left(B\right)\geq {\hat{q}}_{it}\left(B^{+}\right)$ at any end of time *t*. ☐

**Lemma** **2.** At any end of time slot t, the learned expected service quality ${\hat{q}}_{jt}\left(B\right)$ of user j(j∈N\{i}) with bid vector Bis not higher than ${\hat{q}}_{jt}\left(B^{+}\right)$ with bid vector $B^{+}$, that is ${\hat{q}}_{jt}\left(B\right)\leq {\hat{q}}_{jt}\left(B^{+}\right)$.

**Proof.**  As shown in the proof of Lemma 1, we also adopt mathematical induction to prove this lemma.

From the proof of Lemma 1, it is easy to see ${\hat{q}}_{j0}\left(B\right)={\hat{q}}_{j0}\left(B^{+}\right)$. Assume the lemma is true at the end time $t=t_{k}-1\left(t_{k}-1\geq 0\right)$. Now, we need to show ${\hat{q}}_{jt_{k}}\left(B\right)\leq {\hat{q}}_{jt_{k}}\left(B^{+}\right)$ when user *j* is in the system at time $t_{k}$.

- C1: From the proof of Lemma 1, we can see, ${\hat{q}}_{jt_{k}}\left(B\right)\geq {\hat{q}}_{jt_{k}}\left(B^{+}\right)$.
- C2: User *j* can be selected if user *j* is the designated user and remains in the active set. As shown in the proof of Lemma 1, user *j*’s probability of being randomly designated with bid vector *B* is equal to that with bid vector $B^{+}$. Now, we compute user *j*’s probability of being in the active set. Since users join the system dynamically, user *j* may meet with user *i* at time $t_{k}$. If user *j* and user *i* come across at time $t_{k}$, the ranking index of user *j* with bid vector *B* is not smaller than that with bid vector $B^{+}$ due to $b_{i}<b_{i}^{+}$. If user *j* does not meet with user *i* at time $t_{k}$, the ranking index of user *j* with *B* equals that with $B^{+}$. Therefore, user *j*’s probability of staying in the active set with *B* is not higher than that with $B^{+}$. From the above analysis, we can see $P_{jt_{k}}\left(B\right)\leq P_{jt_{k}}\left(B^{+}\right)$. Since the service quality increases with the updated number and ${\hat{q}}_{j(t_{k}-1)}\left(B\right)\leq {\hat{q}}_{j(t_{k}-1)}\left(B^{+}\right)$, we can derive ${\hat{q}}_{jt_{k}}\left(B\right)\leq {\hat{q}}_{jt_{k}}\left(B^{+}\right)$ at the end of time $t=t_{k}$.

Therefore, ${\hat{q}}_{jt_{k}}\left(B\right)\leq {\hat{q}}_{jt_{k}}\left(B^{+}\right)$. To sum up, the lemma is true at any end of time *t*. ☐

**Lemma** **3.** At any time slot t, user i’s probability of being selected with bid vector B is not lower than that with bid vector $B^{+}$, that is $P_{it}\left(B\right)\geq P_{it}\left(B^{+}\right)$.

**Proof.**  Mathematical induction is utilized to prove this lemma. It is easy to see that the lemma is true at time t=0, that is $P_{i0}\left(B\right)\geq P_{i0}\left(B^{+}\right)$.

Assume the lemma is true at the end time $t_{k}-1\left(t_{k}-1\geq 0\right)$. Now, we need to show $P_{it_{k}}\left(B\right)\geq P_{it_{k}}\left(B^{+}\right)$. As the description of Algorithm 1, there are three cases C1, C2 and C3 that user *i* can be selected. For the first two cases C1 and C2, the lemma is true, as we have proven in the Lemma 1.

Assume user *i* is in location $l_{j}$ at time $t_{k}$. For case C3, user *i* can be selected if the following three conditions are satisfied simultaneously.

- User *g* ($g\in N_{jt_{k}}\backslash i$) is the designated one, but user *g* is not in active set $S_{jt_{k}}$. As shown in the case C2 of Lemma 2, user *g*’s probability of being in active set with *B* is not lower than that with $B^{+}$.
- User *i* is in active set $S_{jt_{k}}$. As shown in the case C2 of Lemma 1, user *i*’s probability of being in $S_{jt_{k}}$ with *B* is not lower than that with $B^{+}$.
- User *i* has the highest estimated price-quality ratio among active users in $S_{jt_{k}}$. Since ${\hat{q}}_{it_{k}}\left(B\right)\geq {\hat{q}}_{it_{k}}\left(B^{+}\right)$ is known from Lemma 1 and $b_{i}<b_{i}^{+}$, we can derive ${\hat{q}}_{it_{k}}\left(B\right)/b_{i}\geq {\hat{q}}_{it_{k}}\left(B^{+}\right)/b_{i}^{+}$. For any other competitor *g* ($g\in N_{jt_{k}}\backslash i$), we can derive ${\hat{q}}_{gt_{k}}\left(B\right)/b_{g}\leq {\hat{q}}_{gt_{k}}\left(B^{+}\right)/b_{g}$ due to ${\hat{q}}_{gt_{k}}\left(B\right)\leq {\hat{q}}_{gt_{k}}\left(B^{+}\right)$, which is known from Lemma 2. Therefore, the probability of having the highest estimated price-quality ratio with bid vector *B* is not lower than that with $B^{+}$.

From the above analysis, $P_{it_{k}}\left(B\right)\geq P_{it_{k}}\left(B^{+}\right)$ is true in the case C3.

Together with the case C1 and the case C2 proven before, we can derive $P_{it_{k}}\left(B\right)\geq P_{it_{k}}^{+}\left(B\right)$. To sum up, the lemma is true for any time *t*. ☐

**Theorem** **1.** The allocation algorithm in TOAM is ex post monotone.

**Proof.**  Following directly from Lemma 3 and the definition of the ex post monotonicity of allocation rule in Definition 6, we can derive that our algorithm in TOAM is ex post monotone. ☐

**Corollary** **1.** Our allocation algorithm produces a truthful-in-expectation mechanism via applying the transformation procedure with probability of perturbation μ.

**Theorem** **2.** Individual rationality in TOAM is guaranteed.

**Proof.**  From the transformation procedure presented in Section 2.3, winners’ payments are not lower than their costs. Thus, at any time slot *t*, the winner’s utility is $u_{it}=p_{it}-c_{i}=1/\mu \left(c_{\max}-b_{i}\right)\geq 0$. If the user is not selected, $u_{it}=0$. Therefore, the user’s individual rationality is ensured since the user’s utility is nonnegative. ☐

**Theorem** **3.** TOAM achieves computational complexity O(nlogn) at each round.

**Proof.**  The allocation algorithm is the most complex step in our incentive. As shown in Algorithm 1, computation complexity is O(nlogn), where O(nlogn) is the computation complexity of ranking with the maximum number of users who are competing for one job. ☐

## 5. Performance Evaluation

### 5.1. Simulation Setup

We evaluate our proposed scheme by programming in C++. Our evaluation is based on real trajectory sets from the Dartmouth College mobility traces [21] and synthetic MIT Campus traces, which are generated by a time-variant community model (TVCM) [22]. Considering the integrity of records and the movement regularities of users, we choose the data from 21 September 2003 to 20 October 2003 with 566 access points (APs) for the former datasetand select the top 100 active users. For the latter traces, we generate 30 days traces of 100 users with 100 virtual APs respectively by using the same parameter settings as in [22] to mimic the real MIT mobile social networks [23]. Each AP is regarded as a location. Users can participate in a sensing job when they arrive at any AP. The platform selects a winner if available to collect sensory data for every AP at every sensing time slot.

Each user’s cost and expected service quality are set as two random parameters that follow a [0, 1] uniform distribution respectively. Considering the dishonest behavior of overstating bidding prices, users can raise their prices by a random percentage premium from 0.01% to 99.99%. Every user’s quality of sensing for one sensing job follows a uniform distribution with the expectation of his or her service quality. The coefficient that transforms service quality to monetary reward *α* is set to two. As the ranges of service and cost are the same, we choose two to balance the influence of the service quality and the bidding price. Moreover, we set the parameter μ=0.02 for sampling bidding prices in TOAM. Evaluation results are averaged over 100 runs.

### 5.2. Comparing Algorithms

We compare TOAM with random and greedy schemes. In the random scheme, the platform randomly chooses a winner if possible to perform a sensing job for every location at every time slot. In the greedy scheme, the platform randomly selects a winner if he or she is new in the system. Otherwise, the platform selects a winner with the highest estimated price-quality ratio among arriving users. In both schemes, the platform updates the learned expected service quality of the winner *i* following ${\hat{q}}_{i}=\max\left(\frac{r_{i}}{n_{i}}+\sqrt{\frac{8\ln t}{n_{i}}},{\hat{q}}_{i}\right)$. In both the random and greedy schemes, the winners get paid as much as they ask.

### 5.3. Performance Comparison

We evaluate the performance of different schemes in four metrics—average total payments, quality level of sensing service, profit of platform and social welfare under two datasets, respectively.

**Figure 3:**
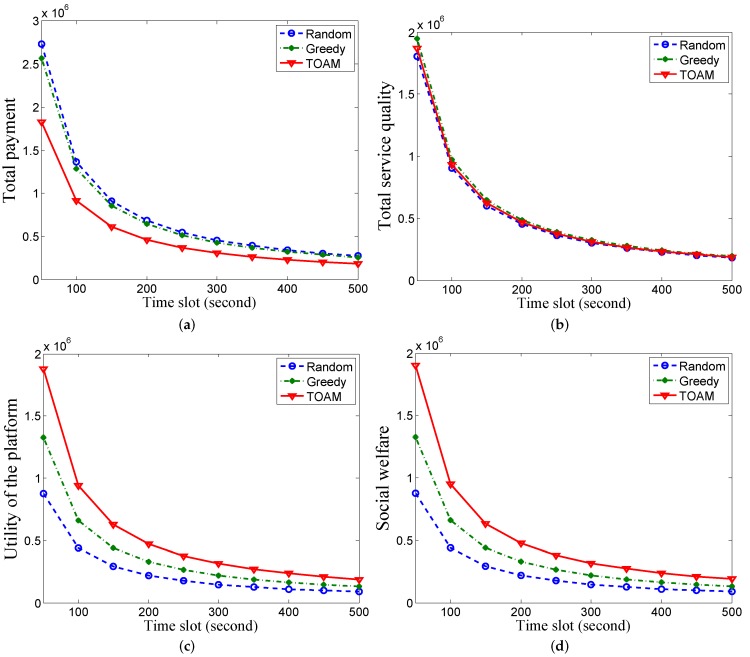
Performance comparisons under the Dartmouth dataset with 100 users. (**a**) Comparison of average payment; (**b**) Comparison of average service quality; (**c**) Comparison of the average utility of the platform; (**d**) Comparison of average social welfare.

We vary the time slot from 50 s to 500 s with an increment of 50 s in the two datasets. Figure 3 shows the results under the Dartmouth trace datasets. In Figure 3a, the average payment of the platform in TOAM is 33.04% and 28.70% on average lower than that in random and greedy, respectively. This is because TOAM ensures that users provide truthful prices (lowest bidding prices), while users overstate prices to pursue higher payments in random and greedy. The payment decreases with the increasing slot gap because the number of sensing jobs is cut down. Since the highest payment of users is fixed, the payment of the platform decreases with less jobs. In Figure 3b, the average quality of sensing in TOAM is 3.36% greater than that in random and 4.04% lower than that in greedy on average. This is because some users with high costs and qualities may be removed when we select a bidding bound with a probability to construct the active user set.

In Figure 3c, the utility of the platform in TOAM is 114.10% and 42.33% greater than that in random and greedy, respectively. This is because TOAM stimulates users to report their truthful costs with similar service qualities comparing with the other two schemes. The social welfare of the sensing system in the TOAM algorithm is 117.98% and 43.89% greater than that in random and greedy, respectively, in Figure 3d. As users are overpaid from the platform as rewards for submitting truthful prices, the increase is a little greater than that in Figure 3c.

**Figure 4:**
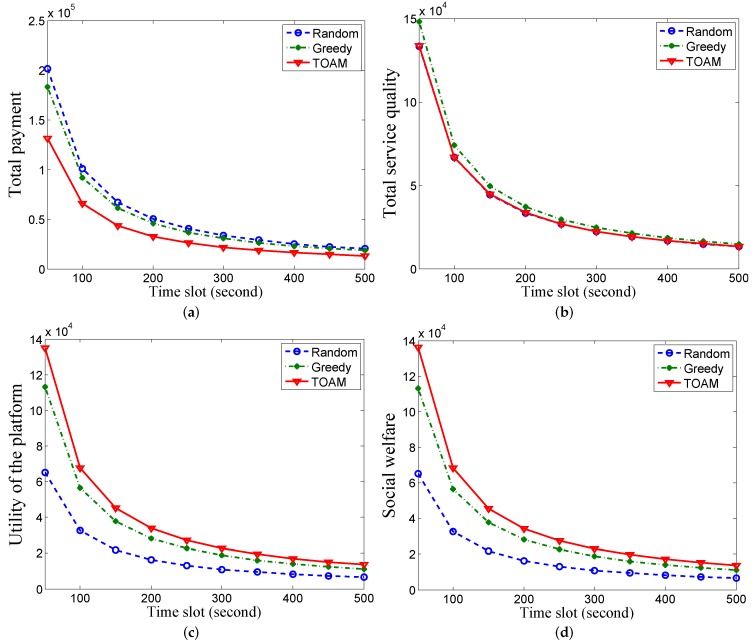
Performance comparisons under the MIT dataset with 100 users. (**a**) Comparison of average payment; (**b**) Comparison of average service quality; (**c**) Comparison of the average utility of the platform; (**d**) Comparison of average social welfare.

Figure 4 shows the results under synthetic MIT traces. Though the density of users in one AP is greater than that in Dartmouth, the results are similar to Figure 3 under Dartmouth traces. As these figures show similar trends, we omit other details.

## 6. Related Work

At first, we review some incentive mechanisms for MCS systems that consider one or two factors of service quality, truthfulness and dynamically-arriving users respectively. Then, we retrospect some truthful single-parameter mechanisms.

### 6.1. Incentive Mechanisms for the Mobile Crowd Sensing System

In order to encourage users to submit timely event reports with high quality, a differentiated monetary incentive for a city management system is proposed in [24]. To estimate the quality of sensing data, the authors in [25] extend the expectation maximization (EM) algorithm combining maximum likelihood estimation and Bayesian inference. The quality of sensing data is defined as the probability that a participant submits sensing data in a valid interval. In [20], the authors present an auction-based incentive for quality-aware and fine-grained MCS in order to maximize the expected expenditure of the platform, where mobile users submit their declared qualities for subtasks and bidding prices before selection. In the above works, the quality of sensing data is considered; but users are static, and truthfulness is not guaranteed.

An online framework is proposed for an MCS system with stochastic arrivals of sensing requests and the dynamic participation of users in [26]. Based on stochastic Lyapunov optimization techniques combined with the idea of weight perturbation, the platform makes online decisions, including admissions of sensing requests and sensing time purchasing from users. In [27], the authors design a recruitment framework for MCS campaigns in opportunistic networks. In this work, users are selected optimally in order to generate the required space-time paths across the network. In [28], an online task assignment algorithm follows the mobility model of users in MCS systems so as to minimize the average makespan of assigned tasks. While the dynamic participation of users is considered in these work, users’ service qualities and dishonest behaviors are ignored.

In [29], the authors design a truthful incentive based on a combinatorial auction for a participatory sensing system, where the platform announces a set of tasks, and users take subsets of these tasks according to their preferences. The system selects an optimal set of users to complete tasks in order to maximize its utility. In [30], the authors introduce a reverse auction framework to design a truthful incentive considering the dimension of location information. In these works, truthfulness is ensured, but users’ service qualities and dynamic participation are ignored.

In [8], the authors design an incentive mechanism that characterizes both the information quality and timeliness of a specific real-time-sensed quantity simultaneously. Information quality is defined as the probability of the presence or absence of the sensing locations. Therefore, information quality can be sampled from a normal distribution with a variable learned from historical experience. In [9], the authors aim at increasing the quality of data directly and introduce gamification to location-based services. The quality indicator is calculated as the probability that the sensory data are categorized to the wrong category at one specific location. In order to increase the qualities of the spacial coverage of locations, the system shows points to users for encouraging users to move there. Considering users’ differential capabilities and uncontrollable mobility [10], designs an incentive to select a minimum subset of participants to satisfy the quality-of-information requirements of multi-tasks with the limited budget. This optimization problem is converted to a nonlinear knapsack problem. The system selects users with the maximum marginal profit under a limited budget dynamically. Users’ sensing qualities for specific locations are associated with the probabilities of arriving there based on historical records. In [31], the authors utilize users’ daily activities, which are known as a priori information, to design a recruitment scheme for participatory sensing, where the similarities of users’ behaviors are utilized to predict the quality of data. Therefore, the allocation problem becomes a collaborative filtering problem. In these works, users’ service qualities are unknown, and users participate dynamically. However, users’ dishonest behaviors are neglected. That is to say, truthfulness is not guaranteed. Besides, these works rely on historical records to start their incentive. Therefore, these above works cannot apply to a new MCS system.

The authors in [5] treat users’ participation levels as users’ service qualities and formalize a truthful incentive based on auction model. In [6], the authors propose truthful incentive mechanisms based on both single-minded and multi-minded combinatorial auctions with the consideration of the information of users’ qualities. However, users’ qualities are known as a priori information while selecting winners. In [7], a truthful incentive mechanism is designed based on a quality-driven auction with an indoor localization system as an example. A probabilistic model is proposed to evaluate the reliability of the sensing data as the users’ service qualities. In these above works, truthfulness is guaranteed. However, users are static, and users’ service qualities are assumed to be known when the sensing platform selects users. In [32], the authors propose a privacy-preserving reputation system for participatory sensing applications, where users exchange information in a lawful manner, and misbehaving can cause them loose their anonymity. However, we use the payment to design a truthful mechanism in order to guarantee users’ honest behaviors in bidding prices based on auction theory. Moreover, users’ service qualities are known as prior knowledge in [32], while they are uncertain information in our work when the platform selects the winners.

The works in [33,34] are the pioneer works on designing online truthful incentive mechanisms based on online auction. The work in [33] models users’ nature of opportunistically occurring in the crowd sensing areas. In the system, the platform decides whether to select users to undertake tasks when they arrive at the system. The authors in [34] design an online truthful incentive based on an online auction model under a budget constraint with the consideration of users’ dynamic participation. The platform selects a subset of users before a specified deadline in order to maximize the value of service, which is assumed to be a non-negative monotone submodular function. In [35], a task is also assumed to be allocated to one user and can be completed in a single slot. The authors design a near-optimal truthful incentive mechanism for the online task allocation scenario, given uncertain arrivals of tasks, dynamic users and users’ strategic behaviors. In [36], a long-term user participation incentive based on a Lyapunov-based VCG auction is provided for a time-dependent and location-aware participatory sensing system. All of these above works are designed with consideration of the dynamic users and the truthfulness of the mechanisms, but they ignore users’ differential service qualities.

### 6.2. Truthful Single-Parameter Mechanisms

Our work also relates to the single-parameter mechanism. Myerson [18] and Archer et al. [37] state that an allocation rule should be monotone in terms of reported bids for a truthful mechanism with single-parameter users. In [12], a general procedure is proposed to transform an ex post monotone allocation rule into a randomized mechanism for the realization of truthfulness in expectation and individual rationality. Moreover, an ex post monotone allocation rule is proposed with the consideration of the stochastic value of users. Since users are static in this allocation rule, it cannot be applied in our situation. In [38], the authors design an ex post truthful incentive via applying the transformation presented in [12] for the crowd source application, where a series of binary labeling tasks needs to be completed. While users’ service qualities are unknown, users are static in this work. In [19], the authors design an ex post monotone allocation rule and transform it via [12] to achieve a truthful-in-expectation mechanism. In this work, the data on shared routers have dynamic prioritization, and demand models are stochastic. While the rest of the capacity of routers is dynamic, the characteristic of capacity is different from users’ dynamic participation. Different from these previous work, in this paper, we design a novel monotone allocation rule with the consideration of dynamically-arriving users and transform it via [12] to achieve a truthful-in-expectation incentive in MCS systems.

## 7. Conclusions

In this paper, for homogenous sensing tasks in a new MCS system, we propose a truthful incentive TOAM based on online auction theory with the consideration of both uncertain service qualities and dynamically-arriving users. We analyze the three crucial properties in TOAM theoretically, which are truthfulness, individual rationality and computational efficiency. In the future, we will focus on a more complex online MCS system where users can compete for heterogeneous tasks and make proper schedules to undertake a bundle of tasks during a certain time.

## Figures and Tables

**Table 1:** Frequently-used notations.

Notation	Description
*N*, *i*	Set of users and a user.
*n*	Number of users.
*T*, *t*	Deadline and a time slot.
*L*, $l_{j}$	Set of locations and the *j*-th location.
$N_{jt}$	Set of arriving users at a location $l_{j}$ at time slot *t*.
$c_{i}$, $b_{i}$	User *i*’s cost and bidding price.
${\tilde{b}}_{i}$	User *i*’s new bidding price for allocation.
$c_{\max}$, $c_{\min}$	Maximum and minimum cost.
$q_{it}$, $q_{i}$, ${\hat{q}}_{i}$	User *i*’s service quality at time slot *t*, expected service quality and learned expected service quality.
$p_{it}$, $u_{it}$	User *i*’s payment and utility at time slot *t*.
$u_{0}$	Utility of the platform.
$S_{jt}$	Active set of users at location $l_{j}$ at time slot *t*.
$n_{i}$	User *i*’s number of being randomly selected.
$r_{i}$, $r_{it}$	User *i*’s total observed service quality and observed service quality at time slot *t*.
$b_{-i}$	Others’ bidding prices, except user *i*.
$b_{i}^{+}$	User *i*’s alternate bidding price that is higher than $b_{i}$.
*B*	Bid vector of all users with $b_{i}$ and $b_{-i}$.
$B^{+}$	Alternate bid vector of all users with $b_{i}^{+}$ and $b_{-i}$.
${\hat{q}}_{it}\left(B\right)$, ${\hat{q}}_{it}\left(B^{+}\right)$	User *i*’s learned expected service quality at time slot *t* with the bid vector *B* and $B^{+}$.
$P_{it}\left(B\right)$, $P_{it}\left(B^{+}\right)$	User *i*’s probability of being selected at time slot *t* with the bid vector *B* and $B^{+}$.
